# Changes in Biochemical Composition and Nutrient Materials in *Apocynum pictum* Honey During Storage

**DOI:** 10.3390/foods13233790

**Published:** 2024-11-25

**Authors:** Li Jiang, Yanning Gong, Yu Zhao, Wanqing Dong, Leyan Guo, Jiaqi Ju, Nana Su

**Affiliations:** 1 State Key Laboratory of Desert and Oasis Ecology, Xinjiang Institute of Ecology and Geography, Chinese Academy of Sciences, Urumqi 830011, China; jiangli@ms.xjb.ac.cn; 2 College of Life Sciences, Nanjing Agricultural University, Nanjing 210095, China; 2023116018@stu.njau.edu.cn (Y.G.); 2024116014@stu.njau.edu.cn (Y.Z.); 9231010222@stu.njau.edu.cn (W.D.); 9231010219@stu.njau.edu.cn (L.G.); 9231010215@stu.njau.edu.cn (J.J.)

**Keywords:** *Apocynum pictum*, honey, flavonoids, metabolomics, storage

## Abstract

*Apocynum pictum* (*A. pictum*) honey is rich in effective ingredients including flavonoids, terpenes, and alkaloids that are beneficial to human health. In this study, widely targeted metabolomics were used to detect the plant-derived secondary metabolites of the same batch of *A. pictum* honey from 2022 to 2024, in order to explore whether storage time changes the quality of *A. pictum* honey, especially the content of plant-derived secondary metabolites with important health benefits. The results showed that storage time had no significant effect on the content of sugars, proteins, and other major components in *A. pictum* honey. At the same time, we also found that although storage time had an impact on the content of some secondary metabolites such as flavonoids in *A. pictum* honey, the changes in the content of the characteristic active ingredient, hyperoside, in *A. pictum* honey were not significant. These findings suggest that storage time has a minimal impact on the quality of *A. pictum* honey. This study provides a theoretical basis for the rational storage of *A. pictum* honey.

## 1. Introduction

Honey is the nectar obtained by bees from the nectar glands inside or outside the flowers of plants, as well as honey brewed from honeydew or insect metabolites collected from the leaves or stems of plants [1]. Honey is rich in nutrients, containing over 180 essential nutrients for the human body [2]. After analysis, it was found that the main components of honey are glucose and fructose, accounting for about 65% to 80% of the total honey. These soluble sugars can be directly absorbed without going through all digestive organs, so honey can quickly supplement the energy needed by the human body. Next is moisture, accounting for 16% to 25%; sucrose ranks third, not exceeding 5% [3]. The remaining components, with the smallest proportion, are secondary metabolites derived from plants, including flavonoids, terpenes, and alkaloids. Although the overall proportion of these substances is low, they are an important foundation for honey to exert its health benefits [4,5]. Research shows that it is precisely because honey is rich in flavonoids that it plays important roles in regulating spleen and stomach functioning, protecting the cardiovascular system, improving immunity, moistening the lungs, and stopping coughing [6].

Honey contains more than 75% sugar and barely any water, which creates a high osmotic pressure environment that makes it difficult for bacteria and other microorganisms to survive. The high osmotic pressure environment can cause bacteria to die due to a large amount of dehydration, thus inhibiting the growth and reproduction of microorganisms [7]. The quality of honey may change due to changes in storage conditions. Research shows that factors such as storage time, storage temperature, and storage environment pH can all affect the quality of honey [8]. Visquert et al. analyzed the effect of different storage temperatures on the color of raw honey from harvesting to processing. The results showed that as the storage temperature increased, the chromaticity, hue, brightness, and white indicators all decreased differently [9]. The impact of storage time on honey quality has always been a hot research topic. There are studies showing that after room temperature conditions, the antioxidant activity, total polyphenol content, and total flavonoid content of locust honey and hundred-flower honey both decrease with increasing storage time, and the antioxidant activity of locust honey decreases more significantly [10].

*Apocynum pictum* is a sub-shrub of the *Apocynum* genus in the *Apocynaceae* family, widely distributed in arid northwest provinces such as Xinjiang, Qinghai, and Gansu, as well as coastal provinces such as Liaoning and Shandong in saline alkali wastelands, Gobi wastelands, or alluvial plains in China [11]. It has abundant wild and cultivated resources. The economic and medicinal value of *A. pictum* is quite high [12,13]. Not only does it have significant ecological benefits in windbreak and sand fixation, water conservation, and improving the ecological environment, it also has significant edible and medicinal value [14,15]. *A. pictum* leaves are rich in flavonoids, organic acids, polysaccharides, proteins, polyphenols, and minerals [16]. These chemical components play a role in antidepressant, anxiolytic (anti-anxiety), anti-obesity, and free radical scavenging activities [16,17,18]. Previous studies have shown that flavonoids are important secondary metabolites of *Apocynum pictum* [19]. They are the primary component responsible for exerting the effects [20]. In addition to the leaves of *A. pictum*, due to the abundant glandular tissue, the production of *A. pictum* honey is also very high. Research has shown that *A. pictum* honey is also rich in a large amount of plant-derived secondary metabolites, especially hyperoside, which is a characteristic class of substances in *A. pictum* honey [21,22].

In order to investigate the effect of storage time on the quality of *A. pictum* honey, this study sampled the same batch of *A. pictum* honey in 2022, 2023, and 2024, and qualitatively and quantitatively analyzed the components of the honey using widely targeted metabolomics. Through this method, we hope to provide some suggestions and a theoretical basis for the optimal storage time of *A. pictum* honey.

## 2. Materials and Methods

### 2.1. Acquisition and Storage of A. pictum Honey

The beehive used for collecting honey from *A. pictum* is placed in Yuli, Xinjiang, China. On 25 August 2022, *A. pictum* honey was collected and subsequently sent for metabolomic analysis. Samples were taken again for testing on 25 August 2023, and 25 August 2024. After metabolomic analysis, the remaining *A. pictum* honey continued to be sealed and stored at room temperature. Three biological replicates were used for each sample.

### 2.2. Metabolites Extraction

Triplicate samples’ extracts were analyzed using a UPLC-ESI-MS/MS system (UPLC, Waters Acquity I-Class PLUS, 34 Maple Street Milford, MA 01757, USA; MS, Applied Biosystems QTRAP 6500+, 500 Old Connecticut Path Framingham, MA 01701, USA). The analytical conditions were as follows, UPLC: column, Waters HSS-T3 (1.8 µm, 2.1 mm × 100 mm); the mobile phase consisted of solvent A, pure water with 0.1% formic acid, and 5 mM Ammonium acetate, and solvent B, acetonitrile with 0.1% formic acid. Sample measurements were performed with a gradient program that employed the starting conditions of 98% A and 2% B and maintained these for 1.5 min. Within 5.0 min, a linear gradient to 50% A and 50% B was programmed. Within 9.0 min, a linear gradient to 2% A and 98% B was programmed, and a composition of 2% A and 98% B was maintained for 1 min. Subsequently, a composition of 98% A and 2% B was adjusted to within 1 min and maintained for 3 min. The flow velocity was set as 0.35 mL per minute, the column oven was set to 50 °C, and the injection volume was 4 µL. The effluent was alternatively connected to an ESI-triple quadrupole-linear ion trap (QTRAP)-MS.

### 2.3. LC-MS/MS Analysis

The ESI source operation parameters were as follows: source temperature 550 °C; ion spray voltage (IS) 5500 V (positive-ion mode)/−4500 V (negative-ion mode); ion-source gas I (GSI), gas II (GSII), and curtain gas (CUR) set at 50, 55, and 35 psi, respectively; collision-activated dissociation (CAD) set to medium. Instrument tuning and mass calibration were performed with 10 and 100 µmol/L polypropylene glycol solutions in QQQ and LIT modes, respectively. QQQ scans were acquired as MRM experiments with the collision gas (nitrogen) set to medium. DP (declustering potential) and CE (collision energy) for individual MRM transitions were conducted with further DP and CE optimization. A specific set of MRM transitions were monitored for each period according to the metabolites eluted within this period.

### 2.4. Quality Control

Principal component analysis (PCA) was applied to analyze *A. pictum* honey from different storage times to study how several principal components could elucidate the internal structure between multiple variables while preserving the basic information of the original variable. The log transform and Pareto scaling were mostly utilized to calculate the principal components. The PCA plot analyzes the sample distribution to identify trends, abnormalities, and variability within and across groups based on the original data. The cohesiveness of quality control (QC) samples is a sign of data repeatability and equipment stability. Better instrument stability and increased consistency in the gathered data are reflected in a more cohesive grouping of QC samples.

### 2.5. Data Analysis

After normalizing the original peak area information with the total peak area, a follow-up analysis was performed. Principal component analysis and Spearman correlation analysis were used to judge the repeatability of the samples within the group and the quality control samples. The identified compounds were searched for classification and pathway information in the KEGG, HMDB, and lipidmaps databases. According to the grouping information, to calculate and compare the difference multiples, a *t* test was used to calculate the significance *p* value of the difference in each compound. The R language package ropls was used to perform OPLS-DA modeling, and 200-times permutation tests were performed to verify the reliability of the model. The VIP value of the model was calculated using multiple cross-validation. The method of combining the difference multiple, the *p* value, and the VIP value of the OPLS-DA model was adopted to screen the differential metabolites. The screening criteria were FC > 1, *p* value < 0.05, and VIP > 1. The significance values of the differential metabolites in KEGG pathway enrichment analysis were calculated using a hypergeometric distribution test.

### 2.6. Statistical Analysis

The data from three biological replicates per experimental set-up were obtained. The statistical significance between the control sample and the treated groups was analyzed using an unpaired, two-tailed Student’s *t*-test. The unpaired, two-tailed Student’s *t*-test was used to examine the statistical significance between the treatment groups and the control sample. The statistical application of GraphPad Prism software version 9 (San Diego, CA, USA) was used for all studies. Differences were considered as significant at *p* ≤ 0.05.

## 3. Results

### 3.1. Dynamic Changes in Metabolites in A. pictum Honey Under Different Storage Times

**Figure 1:**
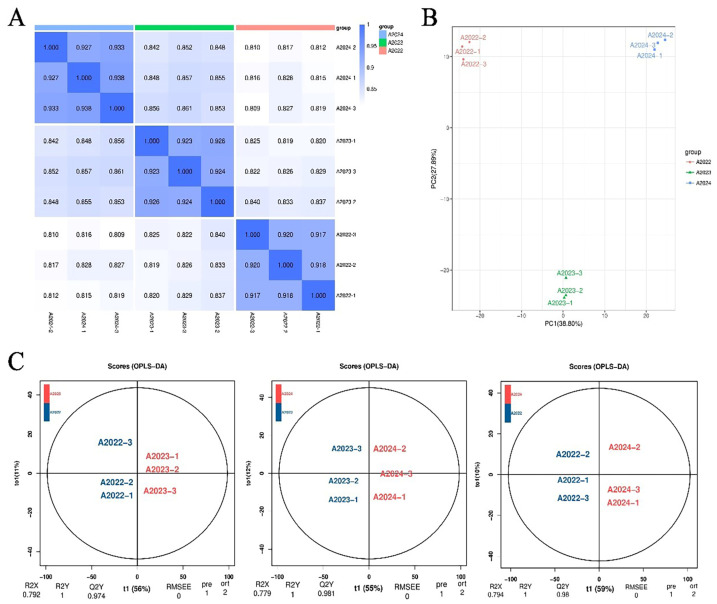
Data quality assessment of widely targeted metabolomics on *Apocynum pictum* honey from 2022 to 2024. (**A**) Correlation chart of widely targeted metabolomics on *A. pictum* honey from 2022 to 2024 samples from 2022 to 2024; (**B**) Widely targeted metabolomics analysis of all samples of *A. pictum* honey from 2022 to 2024 using PCA analysis; (**C**) Widely targeted metabolomics analysis of all samples of *A. pictum* honey from 2022 to 2024 using OPLS-DA analysis.

Samples of the same batch of *A. pictum* honey from the year 2022 to 2024, from *A. pictum* in Yuli, Xinjiang, China (as shown by the red dot in the Figure S1, coordinates 85.54443° N, 41.30025° E), were collected for widely targeted metabolomics research. The characteristic ions of each substance were screened and the signal intensity of the characteristic ions was then obtained. After obtaining metabolite mass spectrometry analysis data from different samples, peak area integration was performed on all substance mass spectrometry peaks, and integration correction was performed on the mass spectrometry peaks of the same metabolite in different samples. Through correlation analysis between samples, it was found that the intergroup correlation coefficients of different samples were all greater than 0.9, indicating high experimental reproducibility (Figure 1A). A total of 1060 metabolites were detected. In terms of classification, they were mainly divided into sugars, amino acids, nucleotides, lipids, vitamins, organic acids, flavonoids, terpenes, alkaloids, polyphenols, and other substances (Table S1). Principal component analysis (PCA) (Figure 1B) and orthogonal projections to latent structures discriminant analysis (OPLS-DA) (Figure 1C) were conducted on these components in *A. pictum* honey over three years. The results showed that, overall, there was no significant difference in the main components of *A. pictum* honey within each group from 2022 to 2024, but there were significant differences between the groups. This indicates that the results are reliable. The differences were not significant within each year, but were mainly present across these three years.

**Figure 2:**
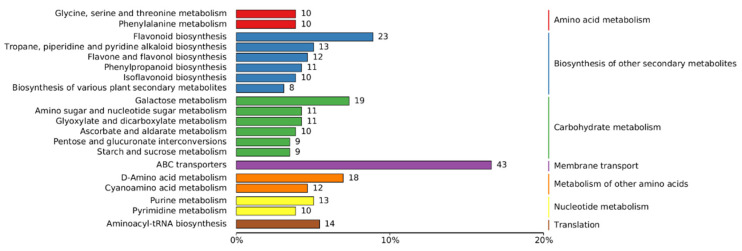
Summary of widely targeted metabolomics KEGG database classification of *A. pictum* honey samples from 2022 to 2024. The 20 pathways with the highest enrichment in KEGG analysis are displayed.

In order to better classify and analyze these metabolites, we annotated the metabolic pathways of these 1060 metabolites using KEGG (Kyoto Encyclopedia of Genes and Genomes) enrichment analysis. The top 20 metabolic pathways with the highest enrichment levels showed that *A. pictum* honey from 2022 to 2024, metabolites were mainly enriched in amino acid metabolism (glycine, serine, and threonine metabolism, phenylalanine metabolism), secondary metabolite biosynthesis (flavonoid synthesis, alkaloid synthesis, and other secondary metabolite synthesis), carbohydrate metabolism (amino sugar and nucleotide sugar metabolism, glyoxylate and dicarboxylate metabolism, ascorbate and aldarate metabolism, pentose and glucuronate interconversions, starch and sucrose metabolism), membrane transporters, other amino acid metabolism, nucleotide metabolism, and translation (Figure 2).

### 3.2. The Effect of Storage Time on the Content of Sugar and Protein Compounds in A. pictum Honey

The highest proportion of sugar substances in honey is various sugars, and another important indicator is protein substances. In order to investigate the effects of different storage times on the content of sugars and proteins in *A. pictum* honey, we conducted detailed statistics on the differential metabolites of sugars and proteins in three groups of comparisons: 2022 vs. 2023, 2023 vs. 2024, and 2022 vs. 2024. We identified 39 sugar compounds with significant changes in content between 2022 and 2023, 35 sugar compounds with significant changes in content between 2023 and 2024, and a total of 34 sugar compounds with significant changes in content throughout the two-year period (2022–2024).

**Figure 3:**
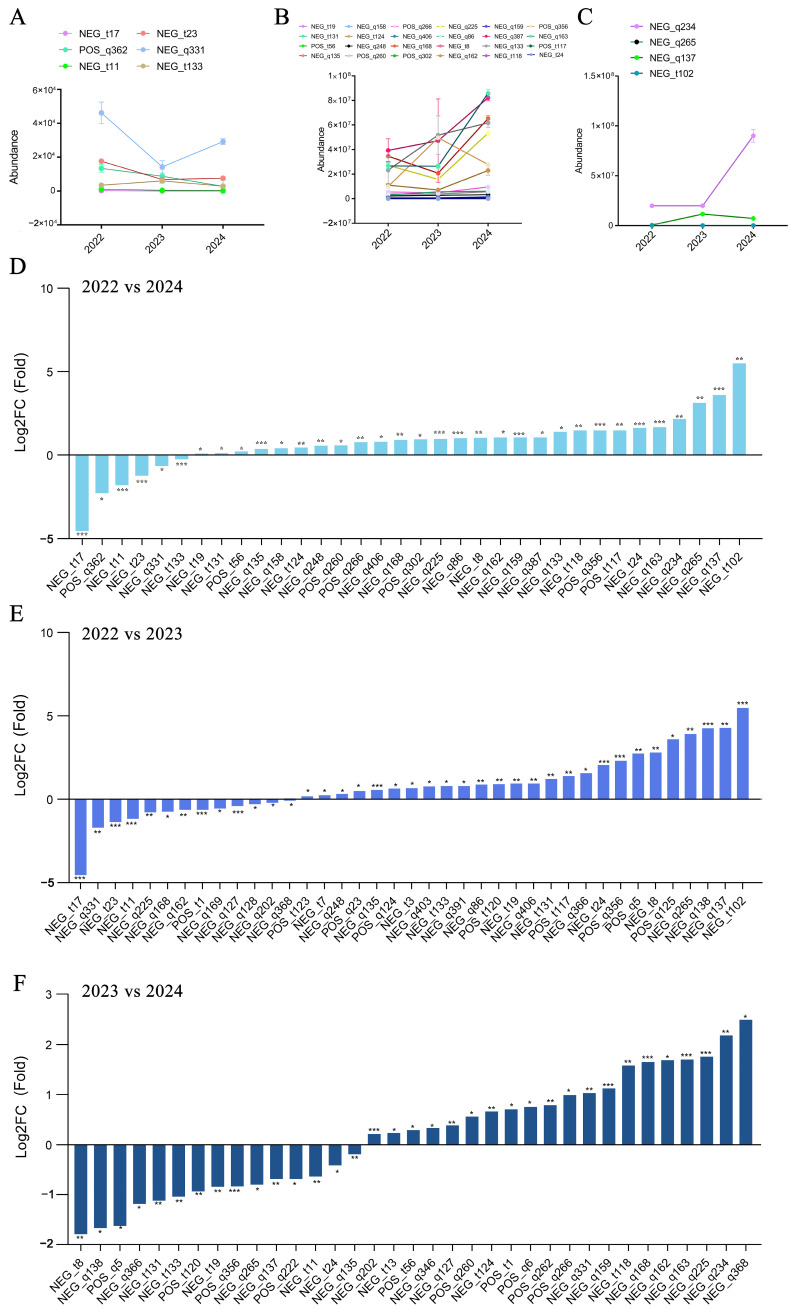
Changes in the content of sugar compounds in *A. pictum* honey from 2022 to 2024. (**A**) Sugar compounds with reduced content in 2024 compared to 2022; (**B**) Sugar compounds with a content increase of less than 2 times in 2024 compared to 2022; (**C**) Sugar compounds with a content increase of more than 2 times in 2024 compared to 2022; The sugar compounds with changes in content in *A. pictum* honey from 2022 to 2024 (**D**), 2022 to 2023 (**E**), and 2023 to 2024 (**F**) are ranked by quantity. The ones with the most significant decreases are placed on the far left of the graph, while the ones with the most significant increases are placed on the far right of the graph. The X axis represents the code of each compound, and the specific compound name referred to can be found in the attached table. Asterisks denote statistically significant differences (* *p* < 0.05; ** *p* < 0.01; *** *p* < 0.001).

To our surprise, with the increase in storage time, there were very few types of sugar compounds that decreased in *A. pictum* honey (Figure 3, Table S2). After one year of storage, the content of 13 sugar compounds decreased, and only rutinose showed a significant decrease. In 2023–2024, there is a decrease in the content of 15 sugar compounds, but the reduction is still very small, with the highest being only 1.79 times. Compared to 2022, the content of only six sugar compounds in *A. pictum* honey has changed in 2024. We believe that the main reason for the decrease in sugar is temperature changes, which lead to honey fermentation and degradation of some of its enzymes. The sugar content of *A. pictum* honey is one of the key factors affecting its storage resistance. A high-sugar environment can form a natural anti-corrosion barrier and inhibit microbial growth. The decrease in sugar content means that this preservative effect is weakened, and *A. pictum* honey is more susceptible to microbial contamination and fermentation deterioration.

**Figure 4:**
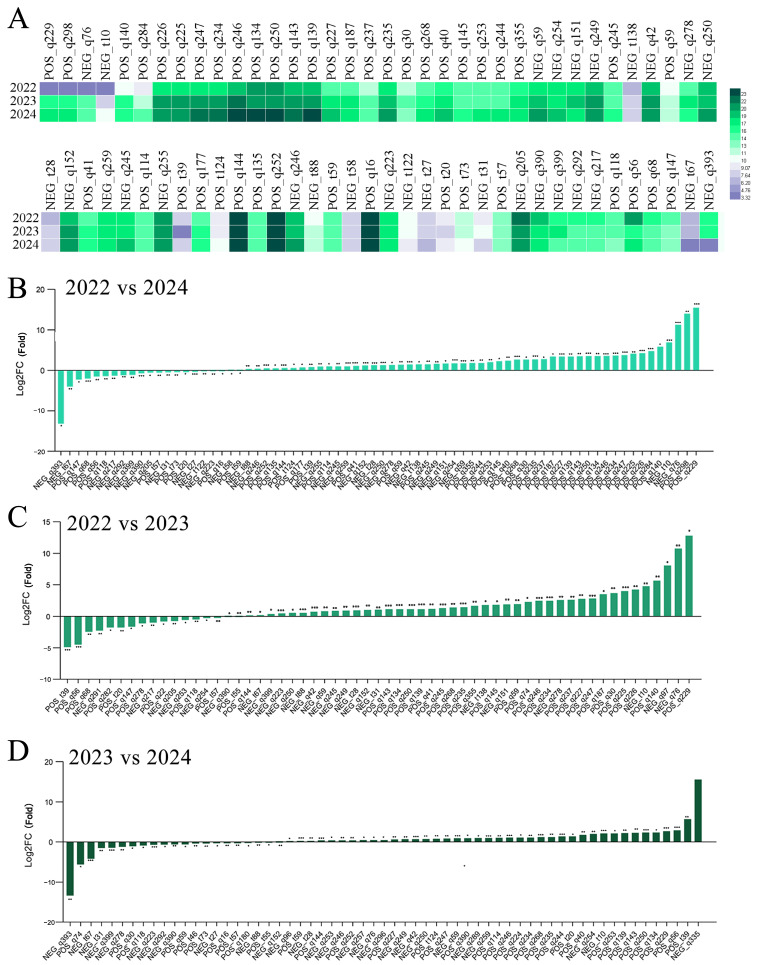
Changes in the content of protein compounds in *A. pictum* honey from 2022 to 2024. Heat map of changes in protein compound content with differences over the three-year period of 2022–2024 (**A**). The protein compounds with changes in content in *A. pictum* honey from 2022 to 2024 (**B**), 2022 to 2023 (**C**), and 2023 to 2024 (**D**) are ranked by quantity. The ones with the most significant decreases are placed on the far left of the graph, while the ones with the most significant increases are placed on the far right of the graph. The X axis represents the code of each compound, and the specific compound name referred to can be found in the attached table. Asterisks denote statistically significant differences (* *p* < 0.05; ** *p* < 0.01; *** *p* < 0.001).

We also conducted statistical analysis on the content of protein compounds (Figure 4, Table S3). Similar to the sugar compounds, the content of some protein compounds has decreased slightly. Between 2022 and 2023, a total of 59 protein compounds underwent changes, with 17 experiencing a decrease in content. However, the content of apratoxin F and adenosine, which showed the greatest reduction, decreased by 4.89 and 4.49 times in 2023 compared to 2022, while the content of the other compounds did not decrease significantly. From 2023 to 2024, a total of 61 protein compounds underwent changes. Among them, 19 compounds decreased. The most significant decrease was observed in uridine 5′-monophosphate, which decreased by 13.38 times. However, the decrease in other compounds was also not significant. Compared to 2022, there was a change in the content of 72 protein compounds. Similarly, a total of 19 compounds showed a decrease in content. But overall, the decrease in the content of these compounds was not significant. Summarizing all the above arguments, it can be inferred that in terms of sugar compounds and protein compounds, as the storage time increases, the content of some of the sugar compounds and protein compounds in *A. pictum* honey changes, but the degree of reduction is not significant.

### 3.3. The Effect of Storage Time on the Content of Flavonoids in A. pictum Honey

In addition to the common compounds, sugar and protein, found in honey, the abundant plant secondary metabolites in honey also endow it with higher health benefits. Among these metabolites, flavonoids play an important role. Meanwhile, due to its high content of flavonoids, *A. pictum* plays a vital role in treating various diseases. Through the KEGG analysis conducted in Section 3.1, it can be found that a large number of metabolites cluster on the flavonoid pathway. Therefore, we proceeded to detect the content of flavonoids in *A. pictum* honey under different storage times.

**Figure 5:**
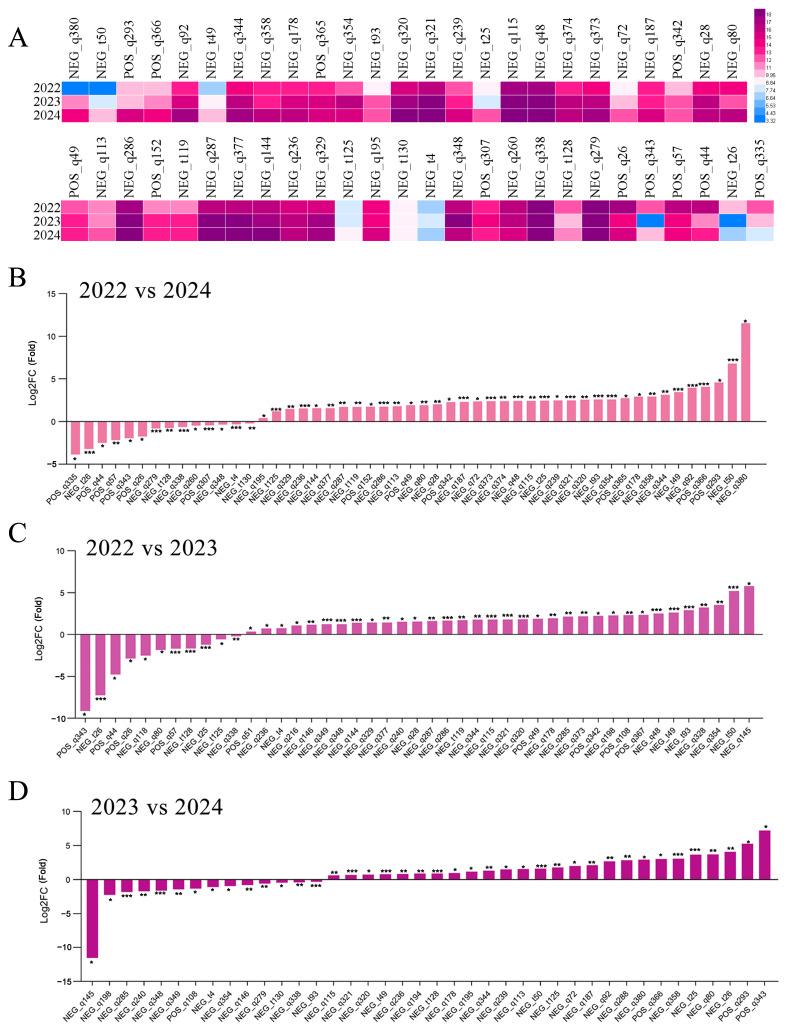
Changes in the content of flavonoids compounds in *A. pictum* honey from 2022 to 2024. Heat map of changes in flavonoid compound content with differences over the three-year period of 2022–2024 (**A**). The flavonoid compounds with changes in content in *A. pictum* honey from 2022 to 2024 (**B**), 2022 to 2023 (**C**), and 2023 to 2024 (**D**) are ranked by quantity. The ones with the most significant decreases are placed on the far left of the graph, while the ones with the most significant increases are placed on the far right of the graph. The X axis represents the code of each compound, and the specific compound name referred to can be found in the attached table. Asterisks denote statistically significant differences (* *p* < 0.05; ** *p* < 0.01; *** *p* < 0.001).

Similarly, it was found that from 2022 to 2023, a total of 45 flavonoids with changes in content were detected, of which 11 compounds showed a decrease in content (Figure 5, Table S4). Among these flavonoids with reduced content, robinin, camellia A, and 5,7-dihydroxyisoflavone showed significant reductions, reaching 9.14, 7.26, and 4.78 times, respectively. The changes in the remaining compounds such as hyperoside were not significant (Figure S2). Between 2023 and 2024, a total of 40 flavonoids were detected to have undergone changes in their levels. The content of 14 compounds decreased in 2024 compared to 2023. The most significant change was that the content of dihydrodaidzein decreased by 11.54 times compared to 2023. The remaining compounds did not decrease by more than three times. Lastly, we compared the differences in flavonoid content in *A. pictum* honey between 2022 and 2024. As a result, 51 flavonoids with altered content were found, of which 14 had decreased content. But we also found that the highest decrease reached 3.86 times. Finally, we compared the differences in flavonoid content in *A. pictum* honey between 2022 and 2024. As a result, 51 flavonoids with altered content were found, of which 14 had a decreased content. But we also found that the highest decrease reached 3.86 times. And most of the flavonoids with reduced content showed a decrease of less than two times. In summary, with the increase in storage time, the content of some flavonoids in *A. pictum* honey decreases, but the overall decrease in flavonoid content is not significant.

## 4. Discussion

Due to its high content of secondary metabolites including flavonoids, *Apocynum pictum* plays a crucial role in the treatment of various diseases [23]. Whether it is used in related products made from the leaves or honey, its market is expanding nowadays. Although honey has a relatively long storage time, the content of some active ingredients might change during the storage process, leading to a decrease in the health value of honey [24]. *A. pictum* honey products are already very mature, but whether storage time will affect the content of ingredients in *A. pictum* honey remains to be analyzed. We choose room temperature as the storage condition because in most cases, honey is stored at room temperature; some honey may crystallize under refrigeration, which can affect the taste; and the transfer rate of chemical processes in honey under refrigeration is slower, especially at temperatures below 0 °C [25]. Therefore, we utilized broad-target metabolomics to investigate this process.

Compared to traditional methods for detecting a specific component or some kinds of components, the widely targeted metabolomics method is now prioritized due to its ability to highly sensitively qualitatively and quantitatively determine the composition and content of different compounds at a high-throughput level [26]. In recent years, widely targeted metabolomics has also been widely used in studying changes in honey components. Yan et al. investigated the changes in representative compound content during processing from nectar to mature honey through metabolomics [27]. In addition, honey markers of different floral and geographic origins were obtained with a strategy for comparative untargeted metabolomics using ultrahigh-performance liquid chromatography-hybrid quadrupole-orbitrap mass spectrometry. For the analysis of metabolomics related to *A. pictum* honey, research has found that compared with other types of honey, *A. pictum* honey is rich in flavonoids, especially flavonols, and one of the representative compounds is hyperoside [28].

Flavonoids play an important role in people’s daily diet [29]. A large number of studies have reported the active role of flavonoids in medicine. Flavonoids have been proven to have a variety of biological activities such as antibacterial, anti-cancer, antioxidant, nervous system protection, and in the prevention of diabetes and cardiovascular disease [30,31,32]. Previous studies have mainly found that quercetin and kaempferol in flavonoids play a key role, and there are many related research results [33]. Quercetin can inhibit nucleic acid synthesis by suppressing lysozyme, thereby exerting antibacterial effects [34]. Shanna phenol has also been found to prevent lipopolysaccharide-induced acute lung injury by inhibiting the NF–κB signaling pathway [35]. In recent years, the importance of hyperoside has also been increasingly discovered by researchers. Hyperoside is widely present in various plants and has various physiological activities such as anti-inflammatory, blood-pressure lowering, cholesterol lowering, and protective effects on the heart and cerebral blood vessels [36]. Research has shown that among all species that have been counted, the content of hyperoside in *A. pictum* reaches 5000 μg g^−1^, far exceeding other species. In a comparison of five types of honey with unique characteristics in northwest China, the content of hyperoside in *A. pictum* honey was significantly higher than that in other species [28]. Therefore, in this study, we specifically focused on the changes in the content of flavonoids, especially hyperoside, in *A. pictum* honey under different storage times. As a result, it was found that with an increase in storage time, although the content of flavonoids in *A. pictum* honey decreased in some components, it remained generally unchanged. More importantly, we did not notice a decrease in the content of hyperoside. Therefore, from the perspective of flavonoid content, especially hyperoside, storage time has no significant effect on the content of the main medicinal components of *A. pictum* honey (Figure S2).

**Figure 6:**
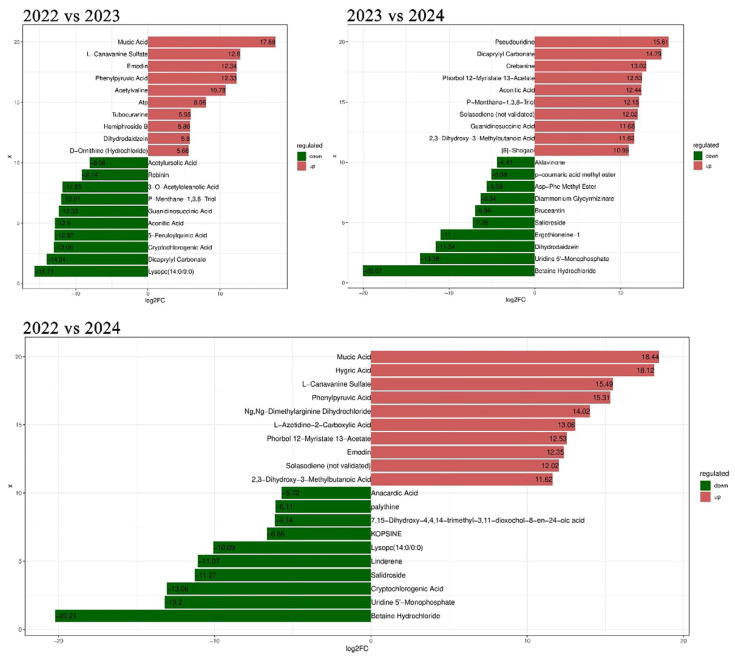
Metabolites with the top 10 highest fold difference between upregulated and downregulated metabolites in *A. pictum* honey from 2022 to 2024.

Although the storage time did not significantly affect the content of major sugar compounds, protein compounds, and flavonoids in *A. pictum* honey, we found that with the increase in storage time, the content of some organic acids (Figure S3, Table S5) and alkaloids (Figure S4, Table S6) in *A. pictum* honey decreased significantly. The sugar content of honey is one of the key factors affecting its storage resistance. There is no significant reduction in the sugar content of *A. pictum* honey, indicating that the nutritional value and flavor of the honey has hardly changed. The reduction in protein means that this antibacterial effect is weakened and the *A. pictum* honey is more likely to spoil. However, the protein content of *A. pictum* honey did not change significantly, indicating that the stability of the honey colloid hardly changed during the two years, and *A. pictum* honey storage tolerance increased. The reduction in organic acids and alkaloids will have an impact on honey preservation. Organic acids can have antibacterial, antioxidant, and other potential active effects in honey, thereby extending the shelf life of honey [37]. There are reports that organic acids can inhibit the growth of pathogenic intestinal bacteria and improve intestinal function [37], and the reduction in alkaloids may reduce the toxicity of honey, but it may also affect its flavor and shelf life [38]. Some alkaloids have also been shown to be promising drugs for managing insulin resistance in recent times [38]. Considering that organic acids and alkaloids in A. *pictum* honey will also have great benefits for people’s health, we recommend consuming *A. pictum* honey as soon as possible while preserving it properly, in order to ensure the stability of the active ingredients in *A. pictum* honey (Figure 6).

The storage conditions and location of honey sources are crucial for the shelf life of honey, as they directly affect the taste and edibility of honey. There is literature indicating that after storing fresh citrus honey at 10, 20, and 40 °C for 12 months, the degree of chemical changes in honey stored at 10 or 20 °C is relatively small, but the Maillard reaction occurs at 40 °C, resulting in significant changes in chemical substances and a shorter storage time [39]. And after 1 year of storage at different storage temperatures, the antioxidant activity, total polyphenol content, and total flavonol content of honey significantly decreased with the extension of storage time, and the higher the temperature, the faster the decrease [9]. Thus, we believe that storing honey at room temperature and in a cool place will greatly extend its shelf life. Of course, as the storage time increases, the total phenolic content in honey may decrease, as total phenols are one of the important antioxidant components in honey. In addition, there are significant differences in the total phenolic content of honey in different regions, which may be related to differences in the types of honey-source plants. This indicates that storage conditions are not the only factor affecting total phenolic content, and plant sources also play an important role. According to previous research, acacia honey from western Romania and manuka honey from New Zealand were stored under the same conditions [40]. The results showed that the total polyphenol and flavonoid content of acacia honey significantly decreased during storage, leading to a decrease in antioxidant capacity and a shortened storage time, while the total polyphenol and flavonoid content of manuka decreased less, greatly increasing its storage time [40]. According to our results, the total polyphenol and flavonoid content of *A. pictum* honey decreased less during storage, indicating that *A. pictum* honey also has a longer storage time and greater medicinal value.

## 5. Conclusions

This study used widely targeted metabolomics to detect changes in the component content of *Apocynum pictum* honey over two years for the first time. The results showed that storage time (within 2 years) had little effect on the sugar and protein compounds in *A. pictum* honey and had no significant effect on the content of the main health-promoting component flavonoids in honey, especially the characteristic compound hyperoside, whose content did not change significantly. These indicated that *A. pictum* honey maintains high stability during its storage. However, considering that the content of some organic acids and alkaloids might significantly change during storage, we strongly recommend consuming *A. pictum* honey as soon as possible; under conditions of proper storage, it can be stored at room temperature, dry, and in a shaded place for up to two years.

## Data Availability

The original contributions presented in this study are included in the article/Supplementary Materials. Further inquiries can be directed to the corresponding author.

## Supplementary Materials

The following supporting information can be downloaded at https://www.mdpi.com/article/10.3390/foods13233790/s1, Figure S1: The location for collecting honey from *Apocynum pictum* to be tested. The red dot with coordinates is the collection point for honey from *A. pictum* in this study; Figure S2: Changes in the content of hyperoside in *A. pictum* honey from 2022 to 2024; Figure S3: Changes in the content of organic acid compounds in *A. pictum* honey from 2022 to 2024. Figure S4: Changes in the content of alkaloids compounds in *A. pictum* honey from 2022 to 2024. Table S1: All metabolites detected in this study; Table S2: Changes in the content of sugar compounds in *A. pictum* honey from 2022 to 2024; Table S3: Changes in the content of protein compounds in *A. pictum* honey from 2022 to 2024; Table S4: Changes in the content of flavonoid compounds in *A. pictum* honey from 2022 to 2024; Table S5: Changes in the content of organic acid compounds in *A. pictum* honey from 2022 to 2024; Table S6: Changes in the content of alkaloids compounds in *A. pictum* honey from 2022 to 2024.

## References

[1] Seraglio S.K.T., Silva B., Bergamo G., Brugnerotto P., Gonzaga L.V., Fett R., Costa A.C.O. (2019). An overview of physicochemical characteristics and health-promoting properties of honeydew honey. Food Res. Int..

[2] Palma-Morales M., Huertas J.R., Rodríguez-Pérez C. (2023). A Comprehensive Review of the Effect of Honey on Human Health. Nutrients.

[3] Sun Y., Liang J., Zhang Z., Sun D., Li H., Chen L. (2024). Extraction, physicochemical properties, bioactivities and application of natural sweeteners: A review. Food Chem..

[4] Hossen M.S., Ali M.Y., Jahurul M.H.A., Abdel-Daim M.M., Gan S.H., Khalil M.I. (2017). Beneficial roles of honey polyphenols against some human degenerative diseases: A review. Pharmacol. Rep..

[5] Jaganathan S.K., Mandal M. (2009). Antiproliferative Effects of Honey and of Its Polyphenols: A Review. BioMed Res. Int..

[6] Nikhat S., Fazil M. (2022). History, phytochemistry, experimental pharmacology and clinical uses of honey: A comprehensive review with special reference to Unani medicine. J. Ethnopharmacol..

[7] Tiencheu B., Nji D.N., Achidi A.U., Egbe A.C., Tenyang N., Tiepma Ngongang E.F., Djikeng F.T., Fossi B.T. (2021). Nutritional, sensory, physico-chemical, phytochemical, microbiological and shelf-life studies of natural fruit juice formulated from orange (*Citrus sinensis*), lemon (*Citrus limon*), Honey and Ginger (*Zingiber officinale*). Heliyon.

[8] Da Silva P.M., Gauche C., Gonzaga L.V., Costa A.C.O., Fett R. (2016). Honey: Chemical composition, stability and authenticity. Food Chem..

[9] Visquert M., Vargas M., Escriche I. (2014). Effect of postharvest storage conditions on the colour and freshness parameters of raw honey. Int. J. Food. Sci. Technol..

[10] Özcan M.M., Ölmez Ç. (2014). Some qualitative properties of different monofloral honeys. Food Chem..

[11] Xie W., Li F., Ding X., Xu Z., Cui Y., Fu X., Xu K. (2024). Ethnomedical Uses, Phytochemistry and Pharmacology of *Apocynum venetum* L. J. Ethnopharmacol..

[12] Jiang L., Wu X., Zhao Z., Zhang K., Tanveer M., Wang L., Huang J., Tian C., Wang L. (2021). Luobuma (*Apocynum*)—Cash crops for saline lands. Ind. Crop Prod..

[13] Xie W., Zhang X., Wang T., Hu J. (2012). Botany, traditional uses, phytochemistry and pharmacology of *Apocynum venetum* L. (Luobuma): A review. J. Ethnopharmacol..

[14] Abubakar A.S., Huang X., Birhanie Z.M., Gao G., Feng X., Yu C., Chen P., Chen J., Chen K., Wang X. (2022). Phytochemical Composition, Antioxidant, Antibacterial, and Enzyme Inhibitory Activities of Various Organic Extracts from *Apocynum hendersonii* (Hook. f.) Woodson. Plants.

[15] Ren H.L., Cao J.M., Chen Y.Y., Li G.-Q. (2008). Current Research State and Exploitation of *Apocynum venetum* L. Northern Hort..

[16] Manzoor M., Muroi M., Ogawa N., Kobayashi H., Nishimura H., Chen D., Fasina O.B., Wang J., Osada H., Yoshida M. (2022). Isoquercitrin from *Apocynum venetum* L. produces an anti-obesity effect on obese mice by targeting C-1-tetrahydrofolate synthase, carbonyl reductase, and glutathione S-transferase P and modification of the AMPK/SREBP-1c/FAS/CD36 signaling pathway in mice in vivo. Food Funct..

[17] Wu T., Li X., Li T., Cai M., Yu Z., Zhang J., Zhang Z., Zhang W., Xiang J., Cai D. (2018). *Apocynum venetum* Leaf Extract Exerts Antidepressant-Like Effects and Inhibits Hippocampal and Cortical Apoptosis of Rats Exposed to Chronic Unpredictable Mild Stress. Evidence-Based Complement. Altern. Med..

[18] Li C., Tan F., Yang J., Yang Y., Gou Y., Li S., Zhao X. (2019). Antioxidant Effects of *Apocynum venetum* Tea Extracts on d-Galactose-Induced Aging Model in Mice. Antioxidants.

[19] Gao G., Chen P., Chen J., Chen K., Wang X., Abubakar A.S., Liu N., Yu C., Zhu A. (2019). Genomic Survey, Transcriptome, and Metabolome Analysis of *Apocynum venetum* and *Apocynum hendersonii* to Reveal Major Flavonoid Biosynthesis Pathways. Metabolites.

[20] Gao G., Abubakar A.S., Chen J., Chen P., Chen K., Yu C., Wang X., Qiu X., Huang X., Shao D. (2023). Comparative genome and metabolome analyses uncover the evolution and flavonoid biosynthesis between *Apocynum venetum* and *Apocynum hendersonii*. iScience.

[21] Shen J., Yang K., Jiang C., Ma X., Zheng M., Sun C. (2020). Development and application of a rapid HPLC method for simultaneous determination of hyperoside, isoquercitrin and eleutheroside E in *Apocynum venetum* L. and *Eleutherococcus senticosus*. BMC Chem..

[22] Zheng M., Liu C., Pan F., Shi D., Zhang Y. (2012). Antidepressant-like effect of hyperoside isolated from *Apocynum venetum* leaves: Possible cellular mechanisms. Phytomedicine.

[23] Chan C., Lau C., Ng Y., Xu L., Chen S., Chan S., Mok D.K. (2015). Discrimination between Leave of *Apocynum venetum* and Its Adulterant, *A. pictum* Based on Antioxidant Assay and Chemical Profiles Combined with Multivariate Statistical Analysis. Antioxidants.

[24] Manickavasagam G., Saaid M., Lim V. (2024). Impact of prolonged storage on quality assessment properties and constituents of honey: A systematic review. J. Food Sci..

[25] Kędzierska-Matysek M., Florek M., Wolanciuk A., Skałecki P. (2016). Effect of freezing and room temperatures storage for 18 months on quality of raw rapeseed honey (*Brassica napus*). J. Food Sci. Technol..

[26] Shen S., Zhan C., Yang C., Fernie A.R., Luo J. (2023). Metabolomics-centered mining of plant metabolic diversity and function: Past decade and future perspectives. Mol. Plant.

[27] Yan S., Mu G., Yuan Y., Xu H., Song H., Xue X. (2024). Exploring the Formation of Chemical Markers in Chaste Honey by Comparative Metabolomics: From Nectar to Mature Honey. J. Agr. Food Chem..

[28] Wang Q., Wei H., Zhou S., Li Y., Zheng T., Zhou C., Wan X. (2022). Hyperoside: A review on its sources, biological activities, and molecular mechanisms. Phytother. Res..

[29] Billowria K., Ali R., Rangra N.K., Kumar R., Chawla P.A. (2024). Bioactive Flavonoids: A Comprehensive Review on Pharmacokinetics and Analytical Aspects. Crit. Rev. Anal. Chem..

[30] Felice M.R., Maugeri A., De Sarro G., Navarra M., Barreca D. (2022). Molecular Pathways Involved in the Anti-Cancer Activity of Flavonols: A Focus on Myricetin and Kaempferol. Int. J. Mol. Sci..

[31] Perez-Vizcaino F., Duarte J. (2010). Flavonols and cardiovascular disease. Mol. Aspects Med..

[32] Federico D., Abin-Carriquiry J.A., Arredondo F., Echeverry C., Rivera-Megret F. (2013). Neuroprotective Actions of Flavones and Flavonols: Mechanisms and Relationship to Flavonoid Structural Features. CNS Agents Med. Chem..

[33] Dabeek W.M., Marra M.V. (2019). Dietary Quercetin and Kaempferol: Bioavailability and Potential Cardiovascular-Related Bioactivity in Humans. Nutrients.

[34] Hosseini A., Razavi B.M., Banach M., Hosseinzadeh H. (2021). Quercetin and metabolic syndrome: A review. Phytother. Res..

[35] Chen X., Yang X., Liu T., Guan M., Feng X., Dong W., Chu X., Liu J., Tian X., Ci X. (2012). Kaempferol regulates MAPKs and NF-κB signaling pathways to attenuate LPS-induced acute lung injury in mice. Int. Immunopharmacol..

[36] Xu S., Chen S., Xia W., Sui H., Fu X. (2022). Hyperoside: A Review of Its Structure, Synthesis, Pharmacology, Pharmacokinetics and Toxicity. Molecules.

[37] Khataybeh B., Jaradat Z., Ababneh Q. (2023). Anti-bacterial, anti-biofilm and anti-quorum sensing activities of honey: A review. J. Ethnopharmacol..

[38] Kaltner F. (2022). Fate of Food-Relevant Toxic Plant Alkaloids during Food Processing or Storing and Analytical Strategies to Unveil Potential Transformation Products. J. Agric. Food Chem..

[39] Castro-Vázquez L., Díaz-Maroto M.C., González-Viñas M.A., de la Fuente E., Pérez-Coello M.S. (2008). Influence of storage conditions on chemical composition and sensory properties of citrus honey. J. Agric. Food Chem..

[40] Hulea A., Obiștioiu D., Cocan I., Alexa E., Negrea M., Neacșu A.-G., Hulea C., Pascu C., Costinar L., Iancu I. (2022). Diversity of Monofloral Honey Based on the Antimicrobial and Antioxidant Potential. Antibiotics.

