# Supplementary figures and images for: Changes in Biochemical Composition and Nutrient Materials in Apocynum pictum Honey During Storage
Source: Foods. 2024 Nov 25;13(23):3790. doi: 10.3390/foods13233790 (PMC11640498; doi:10.3390/foods13233790)

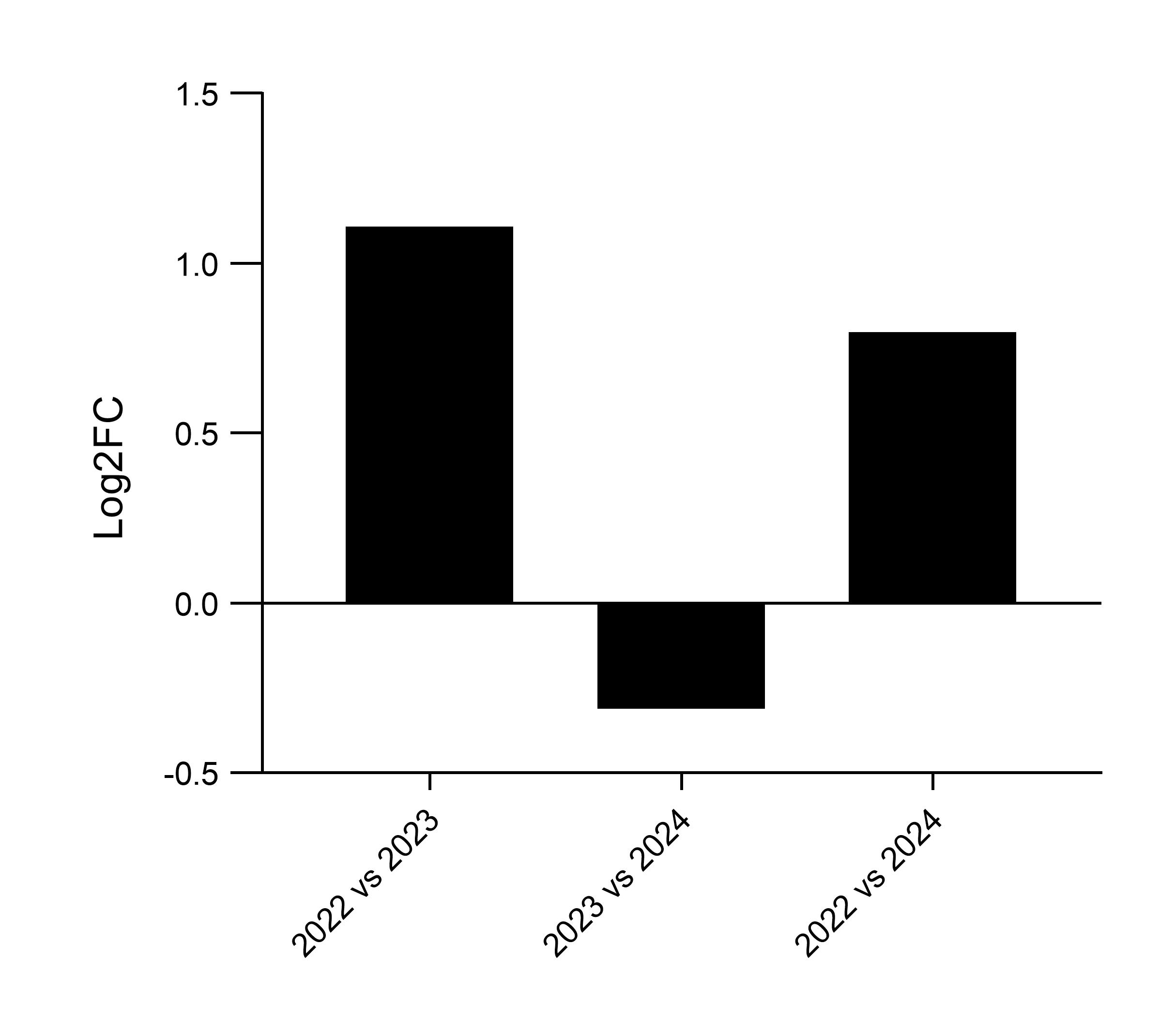

Supplement: Supplementary file 1 [file foods-13-03790-s001.zip › Figure S2.jpg]
